# Advancing rapid diagnostics for bloodstream infections: a perspective on scattered light integrated collection technology

**DOI:** 10.1099/jmm.0.001973

**Published:** 2025-03-12

**Authors:** Shital Shrikant Ghogale, Ketaki Niranjan Pathak

**Affiliations:** 1 Department of Microbiology, Symbiosis Medical College for Women (SMCW) & Symbiosis University Hospital and Research Centre (SUHRC), Symbiosis International (Deemed University), Lavale,, Pune, Maharashtra, India

**Keywords:** bactec, blood culture, patients, scattered light integrated collection (SLIC) device, septicemia, time to positivity

## Dear Editor,

I read with great interest the article by Falconer *et al*. titled *Investigating the Time to Blood Culture Positivity: Why Does It Take So Long?* [1]. The authors have adeptly highlighted a critical issue in clinical microbiology – the inherent delays in detecting bloodstream infections (BSIs) with conventional blood culture (BC) systems. Their exploration of the scattered light integrated collection (SLIC) device provides a promising avenue for overcoming these limitations.

The ability of SLIC to achieve detection from whole blood in significantly reduced time frames, even at low bacterial concentrations, is a noteworthy advancement. This is particularly impactful for BSIs caused by fastidious or slow-growing organisms, which often go undetected in traditional BC systems. Furthermore, the rapid turnaround time of less than 13 h could drastically improve the initiation of targeted antimicrobial therapy, aligning with the critical time-sensitive nature of sepsis management [2,3].

The findings also raise important considerations regarding the suppression of bacterial growth in the presence of blood, as observed with most pathogens in the study. Understanding these dynamics can aid in optimizing pre-analytical protocols and improving detection rates. Moreover, the SLIC device’s potential for point-of-care testing could decentralize BSI diagnostics, making it more accessible in resource-limited settings where laboratory infrastructure is limited [3,4].

There are still several important questions that warrant further exploration. For instance, while SLIC demonstrated promising results with *Escherichia coli*, a broader evaluation with a wide range of pathogens, including anaerobes and multidrug-resistant organisms, is crucial for its clinical applicability. Additionally, the detection of polymicrobial infections and their impact on SLIC’s performance should be explored, as these represent a small yet clinically significant fraction of BSIs [3,4].

It is also essential to evaluate SLIC’s performance in differentiating between true BSIs and contaminants, an area where traditional BC systems often fall short. Integration with existing laboratory information systems and workflows would further determine its feasibility for routine use in clinical microbiology laboratories [4].

The authors’ work paves the way for a paradigm shift in BSI diagnostics. Future studies addressing scalability, cost-effectiveness and robustness across diverse clinical settings will be instrumental in translating this innovative technology into routine practice [5].

## Abbreviations

BC: blood culture
BSIs: bloodstream infections
SLIC: scattered light integrated collection
